# In Utero Exposure to *trans*-10, *cis*-12 Conjugated Linoleic Acid Modifies Postnatal Development of the Mammary Gland and its Hormone Responsiveness

**DOI:** 10.1007/s10911-021-09499-y

**Published:** 2021-10-06

**Authors:** Grace E. Berryhill, Julia M. Gloviczki, Josephine F. Trott, Jana Kraft, Adam L. Lock, Russell C. Hovey

**Affiliations:** 1 Department of Animal Science, University of California, Davis , 2145 Meyer Hall, Davis, CA 95616-8521 USA; 2 Department of Animal and Veterinary Sciences, University of Vermont, Burlington, VT 05405-0148 USA; 3 Department of Animal Science, Michigan State University, East Lansing, MI 48824-1225 USA

**Keywords:** Mammary gland, Conjugated linoleic acid, In utero exposure, Estrogen, Progesterone

## Abstract

We previously showed that dietary *trans*-10, *cis*-12 conjugated linoleic acid (10,12 CLA) stimulates estrogen-independent mammary growth in young ovariectomized mice. Here we investigated the effects of in utero or postnatal exposure to *cis*-9, *trans*-11 (9,11 CLA) and 10,12 CLA on postnatal development of the mammary gland and its responsiveness to ovarian steroids. In the first experiment we fed dams different CLA prior to and during gestation, then cross fostered female pups onto control fed dams prior to assessing the histomorphology of their mammary glands. Pregnant dams in the second experiment were similarly exposed to CLA, after which their female pups were ovariectomized then treated with 17β-estradiol (E), progesterone (P) or E + P for 5 days. In a third experiment, mature female mice were fed different CLA for 28 days prior to ovariectomy, then treated with E, P or E + P. Our data indicate that 10,12 CLA modifies the responsiveness of the mammary glands to E or E + P when exposure occurs either in utero, or postnatally. These findings underline the sensitivity of the mammary glands to dietary fatty acids and reinforce the potential for maternal nutrition to impact postnatal development of the mammary glands and their risk for developing cancer.

**Supplementary Information:**

The online version contains supplementary material available at 10.1007/s10911-021-09499-y.

## Introduction

The in utero environment impacts postnatal development, organ function, and susceptibility to disease [1, 2] and can influence aspects of adult health and wellness including metabolic disorders [3], cardiovascular disease [4], and psychiatric illness [5]. Similarly, in utero exposure to endocrine disruptors [6, 7] and certain nutrients including fats [8] has been linked to aberrant mammary development and susceptibility to developing mammary cancer.

Development of the mammary glands begins in utero with patterning of the milk lines, which, in mice, give rise to 5 placode pairs that develop into individual glands along the mammary ridge [9]. Cross-talk between the epithelial anlagen and the subtending mesenchyme influences formation of the primitive ductal sprout that lies embedded in a well-developed fat pad by the end of gestation [9]. Thereafter the mammary glands grow isometrically until the onset of puberty when ovary-derived estrogen (E), acting through the E receptor (ESR), stimulates rapid expansion of the ductal network as guided by epithelial cell proliferation and resultant terminal end bud (TEB) proliferation and migration [10].

Dietary fats can affect normal development of the mammary glands, and these responses can increase susceptibility of the mammary epithelium to carcinogenesis. Indeed, female offspring of rats fed a high-fat diet (HFD) during pregnancy had more TEB during puberty [8], which increased their postnatal susceptibility to mammary tumorigenesis [11]. Dietary fats can also modify responsiveness of the mammary glands to E, where a HFD increased ESR expression in the mammary epithelium of adult mice, whereas ESR expression was decreased in the mammary glands of females born to dams fed a HFD during gestation [12].

Conjugated linoleic acids (CLA) are 18:2 fatty acids with conjugated double bonds in a *cis* and/or *trans* configuration. These fatty acids occur naturally in meat and milk from ruminants, predominantly as the *cis*-9, *trans*-11 (9,11 CLA) isomer [13], whereas industrial *trans*-hydrogenation of vegetable oils yields multiple CLA isomers including *trans*-10, *cis*-12 (10,12 CLA). Dietary 10,12 CLA dramatically reduces adiposity in rodents [14, 15], leading to its widespread marketing as a weight loss supplement, where up to 8 g/day CLA (a 50:50 mixture of 9,11 and 10,12 CLA) was consumed by clinical trial participants [16]. This class of fatty acids also has a broad range of other biological effects, including on mammary development. Specifically, we identified that dietary 10,12 CLA stimulated allometric growth of the mammary ducts, independent of E and ESR [15]. Female mice fed 10,12 CLA also underwent precocious lobulo-alveolar development [17], increased TEB formation [18], and enhanced oncogene-induced tumorigenesis [15, 19] in their mammary glands. In parallel, diets containing a mixture of 9,11 and 10,12 CLA reduced the burden of mammary tumors in rats administered the chemical carcinogen 7,12-dimethylbenz[a]anthracene [20]. Given these effects of dietary 10,12 CLA and the effects of dietary fat during gestation on the risk for subsequent mammary tumorigenesis [21], we hypothesized that exposure of female mice to CLA in utero would alter postnatal development of the mammary glands and their responsiveness to the ovarian steroid hormones. To this end, we fed mice 9,11 CLA or 10,12 CLA during gestation and evaluated mammary gland development in female offspring born to these dams, as well as the response of their mammary glands to E and progesterone (P). Given that the mammary placodes in fetal mice develop beyond day 10 of gestation [22], we also compared the effect on progeny exposed to maternal dietary 10,12 CLA between either days 1–10 or 11–21 of gestation, relative to those exposed across the entire gestation. Our data reveal that in utero exposure to CLA increases the density of ductal branching in the mammary glands, and enhances the mammary gland responsiveness to E. These findings highlight an effect of the maternal diet during gestation on mammary gland development during crucial periods, such as during the pubertal transition.

## Materials and Methods

### Animals and Diets

All experiments were approved by the University of California, Davis Institutional Animal Care and Use Committee. Balb/cJ mice (Jackson Laboratories, Bar Harbor, ME) had ad libitum access to food and water and were housed under a 14 h light:10 h dark cycle. All diet formulations were based on a modified AIN93G control diet (Harlan Laboratories, Indianapolis, IN, 15% fat supplied primarily as soybean oil). Experimental diets were the control diet with 1% fat (by weight) replaced with 9,11 or 10,12 CLA. The content of 9,11 and 10,12 CLA in the experimental diets was 6.39 and 6.81% of total fatty acids (Table 1), which was 0.96% and 1.02% of the entire 9,11 CLA and 10,12 CLA diets, respectively. As the result of expected and typical partial impurity, the 9,11 CLA diet also contained 0.99% of total fatty acids as 10,12 CLA, while the 10,12 CLA diet also contained 1.19% of total fatty acids as 9,11 CLA (Table 1). No CLA was measurable in the control diet (Table 1).

**Table 1:** Fatty acid profiles (g/100 g of total fatty acids) of experimental diets

	Diet		
Fatty acid	Control	9,11 CLA	10,12 CLA
Σ SFA	15.87	14.95	15.31
16:0	10.98	10.18	10.49
18:0	3.81	3.63	3.79
Σ MUFA	22.11	21.85	20.59
18:1 c9	20.62	20.42	19.25
Σ PUFA	60.77	54.24	54.31
18:2 n6	54.15	48.25	48.36
18:3 n3	6.60	5.95	5.92
Σ CLA	0.00	7.80	8.47
9,11 CLA	0.00	6.39	1.19
10,12 CLA	0.00	0.99	6.81
Σ TFA	0.42	0.34	0.35

*CLA* conjugated linoleic acid, *MUFA* monounsaturated fatty acids, *PUFA* polyunsaturated fatty acids, *SFA* saturated fatty acids, *TFA* trans-fatty acids excluding CLA trans fatty acids, *Σ* Sum of fatty acid class, *cis*-9,* trans*-11 conjugated linoleic acid (9,11 CLA), *trans*-10, *cis*-12 conjugated linoleic acid (10,12 CLA)

### Maternal Transfer of Fatty Acids

In a preliminary pilot study to establish whether CLA were transferred across the placenta, eight-week old female mice were mated, and upon detection of a seminal plug (day 1 of gestation), were assigned to one of the following diet regimens: (1) control diet (n = 2), or that with 1% fat replaced by either (2) 9,11 (n = 1) or (3) 10,12 CLA (n = 1) for the entire gestation. At day 19 of gestation, dams were euthanized by CO_2_ inhalation followed by exsanguination, and fetuses decapitated.

### Experiment 1—Mammary Development in Female Offspring Born to Dams Fed CLA During Gestation

**Fig. 1:**
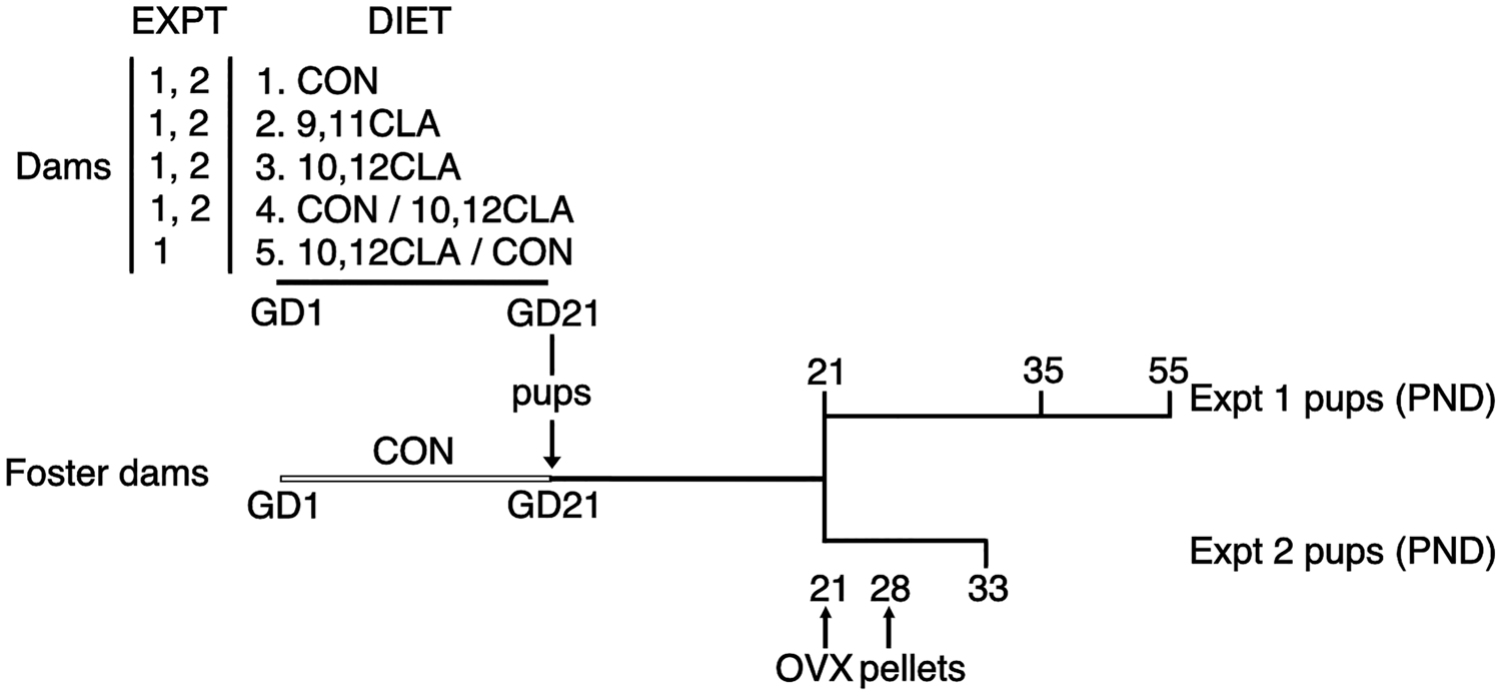
Design and timeline for Experiments 1 (Expt 1) and 2 (Expt 2). At conception, on gestation day 1 (GD1), dams were assigned to consume one of the various diets (either (1) control (CON), (2) *cis*-9, *trans*-11 conjugated linoleic acid (9,11 CLA), (3) *trans*-10, *cis*-12 conjugated linoleic acid (10,12 CLA), (4) control diet from day 0–10 and 10,12 CLA diet from day 10 of gestation until parturition (CON/10,12 CLA) or (5) 10,12 CLA from 0–10 days of gestation and control diet from day 10 of gestation until parturition (10,12 CLA/CON)). Pups born to experimental dams were cross-fostered onto stage-matched control-fed dams. Pups from Expt 1 were euthanized on postnatal day (PND) 21, 35 or 55. For Expt 2, weaned pups were ovariectomized (OVX) and implanted with steroid hormone pellets (SC) on PND 28, then euthanized on PND 33

Eight-week old female mice were mated and upon detection of a seminal plug (day 1 of gestation) were assigned to one of the following diet regimens: (1) the control diet, or that with 1% fat replaced by either (2) 9,11 or (3) 10,12 CLA for the entire gestation, (4) the control diet for the first 10 days of gestation followed by the 10,12 CLA diet for the remainder of gestation, (5) or the 10,12 CLA diet for the first 10 days of gestation followed by the control diet until parturition (Fig. 1). A cohort of stage-matched control-fed females was used as foster dams. Female pups from all dams were cross-fostered onto control-fed dams as soon as the litter was detected (approx. 6-12 h, and no more than 24 h, after birth). Litter size was standardized to n = 6 pups. Pups gestated by dams in all treatment groups were weaned onto the control diet at 21 days of age. A subset of female pups born to dams in each treatment group was euthanized at 21 or 35 days of age. Another cohort was euthanized at 54-59d of age during diestrus, where stage of estrous was determined by vaginal appearance [23] as confirmed by vaginal cytology [24]. Mice were administered 5-bromo-2-deoxyuridine (BrdU; IP, 70 mg/kg; Roche, Mannheim, Germany) for 2 h prior to euthanasia by CO_2_ inhalation, followed by exsanguination (n = 4–9/age group/maternal diet).

### Experiment 2—Hormone Responsiveness of the Mammary Glands in Female Offspring Born to Dams Fed CLA During Gestation

Eight-week old female mice were mated and, upon detection of a seminal plug, were assigned to either the (1) the control diet, or that with 1% fat replaced by either (2) 9,11 or (3) 10,12 CLA for the entire gestation, or (4) the control diet for the first 10 days of gestation followed by the 10,12 CLA diet for the remainder of gestation (Fig. 1). Females were maintained on these diets throughout gestation. Female pups born to dams in each treatment group were cross-fostered onto stage-matched, control-fed dams as soon as each litter was detected (approx. 6-12 h, and no more than 24 h, after birth), with litters standardized to n = 6 pups. Pups were weaned onto the control diet at 21 days of age. Female pups born to dams in each treatment group were bilaterally ovariectomized (OVX) at weaning under ketamine/xylazine anesthesia (IP, 60/10 mg/kg) followed by buprenorphine (SC, 0.05 mg/kg) analgesic. Following a 7d recovery, mice were implanted (n = 4–8/hormone/diet) with a slow-release hormone pellet (SC, ~ 10 mg) containing either cholesterol excipient alone (Sigma-Aldrich, St. Louis, MO), or supplemented with E (5 μg; 17β-estradiol, Sigma-Aldrich), P (3.33 mg; Sigma-Aldrich), or E + P. Five days after pellet implantation, mice were injected with ethynyl-2′-deoxyuridine EdU (100 μl IP in sterile saline, 12.5 mg/kg) 2 h prior to euthanasia that was performed as outlined for Experiment 1.

### Experiment 3—Hormone Responsiveness of the Mammary Glands in Sexually Mature Female Mice Fed CLA

Sexually-mature female mice (56-70d of age) were fed either the control diet or that containing 9,11 or 10,12 CLA for 28d before they were OVX as described above. After a period of 7d to clear endogenous ovarian hormones, mice were administered daily injections of vehicle (Veh, SC, 100 μl corn oil), E (1 μg/d 17β-estradiol), P (1 mg/d), or E + P, for 4d. On the final day of hormone treatment mice were administered BrdU (IP, 70 mg/kg) 2 h before euthanasia by carbon dioxide (CO_2_) inhalation prior to euthanasia (n = 4–13/hormone/diet), as described above.

### Mammary Gland Whole Mount Preparation and Analysis

A single fourth inguinal mammary gland was collected from each mouse and whole mounted as described [25] prior to digital imaging using a stereoscope (Olympus SZX16, Shinjuku, Tokyo, Japan). Ductal elongation was measured as the distance from the teat to the farthest-reaching ductal terminus. Ductal area was defined as the polygon area outlining the ductal network [15] while branchpoint density was expressed as the total number of ductal bifurcations normalized to the total ductal area. Ductal thickness was determined by calculating the average diameter of all ducts that intersected a transverse line drawn midway between the teat and the lymph node. All quantitation of images was performed using NIH ImageJ [26].

### Histology

Left and right thoracic mammary glands (2nd and 3rd) were fixed overnight in 4% paraformaldehyde (Experiment 1) or Tellyesniczky’s Fixative (Experiment 2), dehydrated, and embedded in paraffin. Whole mounts prepared from inguinal mammary glands (4th, Experiment 3) were demounted and oriented in paraffin so that the mammary ducts between the teat and the lymph node could be sectioned transversely. Sections (4–5 μm) were stained with either hematoxylin and eosin, or Gomori’s trichrome.

### Immunohistochemistry

Paraffin sections (4–5 μm) of thoracic mammary glands were subjected to immunohistochemistry, either for BrdU using a biotinylated anti-BrdU monoclonal antibody (1:100; RRID:AB_2536438; Invitrogen, Carlsbad, CA) as described [27], or a biotinylated anti-F4/80 monoclonal antibody (1:200; RRID:AB_1102552; Bio-Rad, Hercules, CA) [28]. The only deviation from the previously-published protocols was that the secondary antibody was not biotinylated. In subsequent experiments we used the more convenient method to detect mitotic cells via incorporation of EdU into mitotic cells, as detected using the Alexa 488 fluorochrome (Click-iT Detection Assays, Invitrogen), with nuclear counterstaining using DAPI in mounting medium (VectaShield, Vector Laboratories).

### Fatty Acid Analyses

Analysis of fatty acids in lipids extracted from diets was performed as described [15]. Lipids were extracted from mammary glands and fetuses using a modified Bligh and Dyer [29] technique [30]. Fatty acid methyl esters were produced by transesterification of extracted lipids using methanolic sodium methoxide, then were analyzed using a GC-2010 gas chromatograph (Shimdazu). Individual fatty acids and fatty acid classes were expressed as percentages (wt/wt) of total fatty acid methyl esters detected. The lowest peak detection was < 0.01% area.

### Statistics

Data were analyzed by one- or two-way ANOVA using the Proc GLM procedure in SAS (Cary, NC) or using Prism8 (Graphpad Software), followed by a post-hoc Dunnet or Tukey test while controlling for multiple testing. Data were transformed where appropriate. Least square means comparisons were considered significant at *P* ≤ 0.05.

## Results

### Maternal Transfer of Fatty Acids

To confirm maternal–fetal transfer of CLA we first analyzed the fatty acid profile of whole fetuses at day 19 of gestation that had been carried by dams fed 9,11 or 10,12 CLA during gestation. The level of 9,11 CLA was increased in fetuses from dams fed the 9,11 CLA diet compared to those from dams fed 10,12 CLA or the control diet during gestation (*P* < 0.05, Table 2). Similarly, 10,12 CLA was detected in fetuses gestated by dams fed 10,12 CLA (*P* < 0.05, Table 2). These data confirmed that CLA from the maternal diet is indeed transferred to, and incorporated into, fetal tissues.

**Table 2:** Fatty acid profiles (g/100 g of total fatty acids) of day 19 fetuses gestated by dams fed either the control diet or that with 1% fat as *cis*-9, *trans*-11 or *trans*-10, *cis*-12 conjugated linoleic acid (9,11 or 10,12 CLA, respectively)

Fatty Acid	Control		9,11 CLA		10,12 CLA		*P* value^d^
	Mean	SEM	Mean	SEM	Mean	SEM	
Σ SFA	41.14	0.21	41.39	0.42	41.69	0.26	NS
16:0	26.43	0.19	26.68	0.08	27.12	0.25	NS
18:0	11.98	0.11	11.91	0.07	11.95	0.12	NS
Σ MUFA	19.83^a^	0.17	19.55^a^	0.07	18.77^b^	0.22	*
18:1 9c	13.08^a^	0.08	12.83^a^	0.04	12.27^b^	0.09	***
Σ PUFA	33.92	0.28	33.49	0.10	34.44	0.44	NS
18:2 n6	9.76^a^	0.17	10.27^a^	0.06	11.62^b^	0.19	***
18:3 n3	0.61^a^	0.01	0.65^b^	0.01	0.68^b^	0.01	**
18:3 n6	0.17^a^	0.01	0.17^a^	0.00	0.13^b^	0.00	***
20:4 n6	10.90^a^	0.04	10.45^b^	0.03	9.70^c^	0.11	***
22:6 n3	7.50^a^	0.08	7.14^b^	0.05	7.60^ab^	0.14	*
Σ CLA	0.00^a^	0.00	0.68^b^	0.01	0.49^c^	0.10	***
9,11 CLA	0.00^a^	0.00	0.52^b^	0.01	0.09^c^	0.00	***
10,12 CLA	0.00^a^	0.00	0.04^b^	0.00	0.32^c^	0.01	***

*CLA* conjugated linoleic acid, *MUFA* monounsaturated fatty acids, *PUFA* polyunsaturated fatty acids, *SFA* saturated fatty acids, *Σ* Sum of fatty acid class

*P < 0.05; **P < 0.001; ***P < 0.0001 or NS not significant

^a,b,c^Means with different superscripts are different within a row *P* < 0.05. n = 6–11 fetuses/diet

^d^Main effect of diet

### Experiment 1—Mammary Development in Female Offspring Born to Dams Fed CLA During Gestation

Analysis of fatty acids extracted from the mammary glands of 21, 35, or 55 days-old female mice born to dams fed 9,11 or 10,12 CLA during gestation indicated that the level of CLA in this tissue did not change with age (*P* > 0.05, Table 3). However, there was a significant main effect of diet on the content of 9,11 CLA (*P* = 0.0001, Table 3), where offspring born to dams fed 9,11 CLA had a higher content of this CLA in their mammary glands than did offspring born to the three treatment groups supplemented with 10,12 CLA (*P* < 0.005). The highest % of 9,11 CLA in the mammary glands was in 21-day old mice exposed to 9,11 CLA in utero (Table 3). There was no measurable 10,12 CLA in the mammary glands of mice born to dams fed 10,12 CLA (data not shown). There was also an effect of age on the fatty acid profile of the mammary glands for all lipid classes analyzed, excluding 9,11 CLA (*P* < 0.0001, Table 3). Total content of saturated fatty acids decreased with age, while monounsaturated fatty acid and polyunsaturated fatty acid content increased (*P* < 0.0001, Table 3). The proportion of linoleic acid (18:2n6) similarly increased with age (*P* < 0.0001, Table 3) whereas the content of gamma-linolenic acid (18:3n6), arachidonic acid (20:4n6) and docosahexaenoic acid (DHA, 22:6n3) decreased (*P* < 0.0001, Table 3). There was a significant interaction between diet and age for total saturated fatty acids, palmitic acid (16:0), total monounsaturated fatty acids, total polyunsaturated fatty acids, linoleic acid, and DHA (*P* < 0.05, Table 3).

**Table 3:** Fatty acid profiles of mammary glands from 21, 35 or 55d old female mouse pups in Experiment 1. Dams of pups were fed the CON diet or that with 1% fat as *cis*-9, *trans*-11 or *trans*-10, *cis*-12 conjugated linoleic acid (9,11 or 10,12 CLA, respectively) throughout gestation (d0-parturition), or the control (d0-10) diet followed by the 10,12 CLA diet (d11-parturition), or the 10,12 CLA (d0-10) followed by the control diet (d11-parturtion)

	Maternal diet (feeding period)																*P* values			
	CON (d0-parturition)			9,11 CLA (d0-parturition)			10,12 CLA (d0-parturition)			CON (d0-10) and 10,12 (d11-parturition)			10,12 CLA (d0-10) and CON (d11-parturition)							
Fatty Acid	21d	35d	55d	21d	35d	55d	21d	35d	55d	21d	35d	55d		21d	35d	55d		A	D	A*D
Σ SFA	38.20^a^ (0.86)	24.91^b^ (0.52)	19.79^de^ (0.72)	36.54^a^ (0.94)	22.83^bc^ (1.47)	22.08^ cd^ (0.68)	37.89^a^ (0.31)	24.46^bc^ (0.24)	20.79^cde^ (0.26)	38.84^a^ (0.13)	25.40^b^ (0.40)	19.43^e^ (0.14)		39.07^a^ (0.31)	25.41^b^ (0.75)	18.93^e^ (0.41)		***	NS	***
16:0	21.94^a^ (0.42)	17.04^b^ (0.31)	15.15^ cd^ (0.34)	21.46^a^ (0.31)	16.41^bc^ (0.50)	16.75^b^ (0.38)	21.71^a^ (0.19)	16.81^b^ (0.19)	16.32^bcd^ (0.22)	22.05^a^ (0.17)	17.07^b^ (0.28)	15.06^ cd^ (0.17)		22.05^a^ (0.08)	16.85^b^ (0.41)	14.45^d^ (0.33)		***	NS	***
18:0	1.53^a^ (0.11)	2.54^c^ (0.15)	2.46^c^ (0.11)	1.68^ab^ (0.11)	2.40^c^ (0.18)	2.71^c^ (0.14)	1.43^a^ (0.02)	2.46^c^ (0.10)	2.58^c^ (0.09)	1.47^a^ (0.04)	2.42^c^ (0.09)	2.17^bc^ (0.06)		1.40^a^ (0.04)	2.49^c^ (0.16)	2.28^c^ (0.11)		***	NS	NS
Σ MUFA	25.93^a^ (0.19)	31.39^b^ (0.42)	34.61^d^ (0.74)	25.89^a^ (0.52)	33.70^ cd^ (1.69)	34.79^d^ (0.53)	26.12^a^ (0.27)	31.04^bc^ (0.36)	34.64^d^ (0.61)	25.09^a^ (0.10)	31.07^bc^ (0.33)	35.32^d^ (0.26)		25.31^a^ (0.28)	30.83^b^ (0.40)	35.56^d^ (0.17)		***	NS	*
18:1 9c	19.82^a^ (0.23)	26.32^b^ (0.34)	29.05^c^ (0.67)	20.22^a^ (0.51)	27.84^bc^ (1.27)	28.67^c^ (0.51)	19.83^a^ (0.21)	25.88^b^ (0.30)	28.76^c^ (0.38)	19.30^a^ (0.10)	26.04^b^ (0.34)	29.18^c^ (0.20)		19.39^a^ (0.22)	25.98^b^ (0.50)	29.77^c^ (0.26)		***	NS	NS
Σ PUFA	35.6^a^ (0.71)	43.38^b^ (0.75)	45.34^b^ (0.31)	37.29^a^ (0.57)	43.22^b^ (0.62)	42.87^b^ (0.30)	35.70^a^ (0.47)	44.23^b^ (0.51)	44.28^b^ (0.71)	35.79^a^ (0.11)	43.27^b^ (0.48)	44.99^b^ (0.30)		35.34^a^ (0.18)	43.41^b^ (0.75)	45.24^b^ (0.45)		***	NS	*
18:2n6	29.67^a^ (0.7)	39.01^b^ (0.64)	41.11^bc^ (0.28)	31.29^a^ (0.57)	39.01^bc^ (0.50)	38.81^bc^ (0.31)	29.81^a^ (0.43)	39.67^bc^ (0.47)	40.05^bc^ (0.60)	29.81^a^ (0.07)	38.82^b^ (0.48)	40.72^bc^ (0.25)		29.57^a^ (0.15)	38.87^bc^ (0.75)	41.16^c^ (0.41)		***	NS	*
18:3n3	2.21^a^ (0.12)	2.49^abc^ (0.09)	2.80^ cd^ (0.07)	2.33^ab^ (0.06)	2.54^abcd^ (0.06)	2.69^bcd^ (0.04)	2.24^a^ (0.07)	2.64^bcd^ (0.08)	2.85^d^ (0.08)	2.24^a^ (0.03)	2.40^ab^ (0.07)	2.77^ cd^ (0.05)		2.21^a^ (0.04)	2.44^abc^ (0.10)	2.62^bcd^ (0.04)		***	NS	NS
18:3n6	0.31^a^ (0.01)	0.09^bc^ (0.01)	0.06^e^ (0.01)	0.30^a^ (0.01)	0.09^bcd^ (0.01)	0.06^de^ (0.01)	0.30^a^ (0.01)	0.11^b^ (0.01)	0.06^e^ (0.00)	0.30^a^ (0.01)	0.11^b^ (0.01)	0.06^cde^ (0.00)		0.29^a^ (0.01)	0.12^b^ (0.01)	0.06^de^ (0.00)		***	NS	NS
20:4n6	0.75^a^ (0.01)	0.35^b^ (0.02)	0.31^b^ (0.02)	0.74^a^ (0.03)	0.33^b^ (0.03)	0.32^b^ (0.01)	0.73^a^ (0.03)	0.36^b^ (0.01)	0.33^b^ (0.01)	0.73^a^ (0.02)	0.36^b^ (0.01)	0.34^b^ (0.01)		0.69^a^ (0.01)	0.38^b^ (0.02)	0.32^b^ (0.01)		***	NS	NS
22:6n3	0.28^a^ (0.02)	0.15^ cd^ (0.01)	0.17^ cd^ (0.01)	0.27^ab^ (0.01)	0.14^d^ (0.01)	0.14^d^ (0.00)	0.25^ab^ (0.01)	0.14^d^ (0.01)	0.17^ cd^ (0.01)	0.26^ab^ (0.01)	0.13^d^ (0.01)	0.17^ cd^ (0.01)		0.22^bc^ (0.01)	0.15^d^ (0.01)	0.17^ cd^ (0.01)		***	NS	*
9,11 CLA	0.03^ab^ (0.01)	0.03^ab^ (0.00)	0.03^ab^ (0.00)	0.06^b^ (0.01)	0.03^ab^ (0.00)	0.03^ab^ (0.00)	0.02^a^ (0.00)	0.03^ab^ (0.00)	0.02^a^ (0.00)	0.02^a^ (0.00)	0.02^a^ (0.00)	0.03^ab^ (0.00)		0.03^a^ (0.01)	0.02^a^ (0.01)	0.03^ab^ (0.00)		NS	**	NS

Data are mean (SEM), n = 4–8/group

Σ Sum of fatty acid class

*CLA* conjugated linoleic acid, *CON* control, *MUFA* monounsaturated fatty acids, *PUFA* polyunsaturated fatty acids, *SFA* saturated fatty acids

Main effect of age (A), main effect of diet (D), interaction of age and diet (A*D) at * *P* < 0.05; ** *P* < 0.005; *** *P* < 0.0001 or NS not significant

^a,b,c,d,e^Means with different superscripts are different *P* < 0.05

**Fig. 2:**
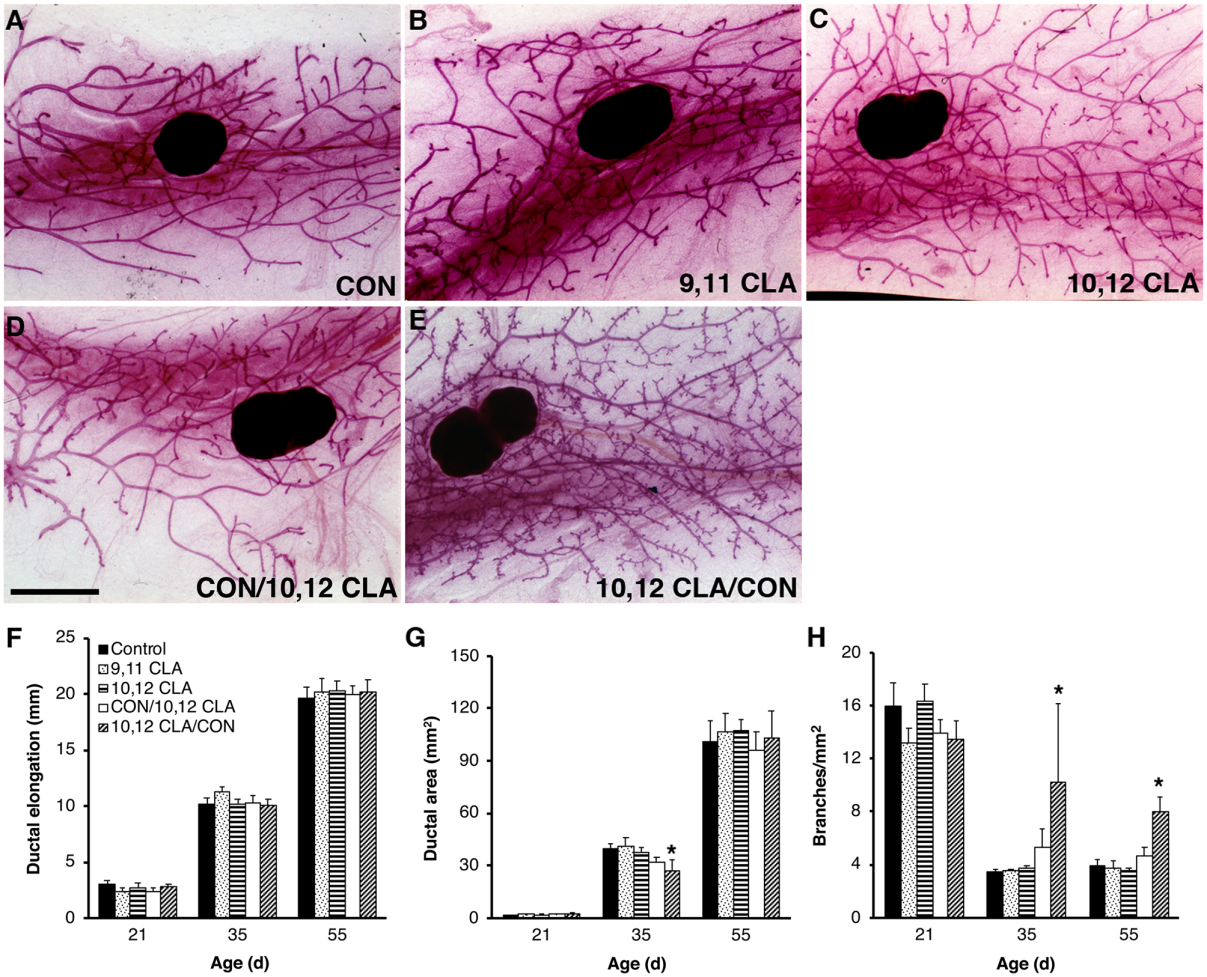
Exposure to 10,12 CLA during early in utero development via the maternal diet increased branching density in the mammary glands of peri- and postpubertal female mice in Experiment 1. **a** − **e** Whole mounts of mammary glands from 55-day old female mice exposed via the maternal diet to either: **a** control (CON) diet, **b** *cis*-9, *trans*-11 conjugated linoleic acid (9,11 CLA) diet, **c** *trans*-10, *cis*-12 conjugated linoleic acid (10,12 CLA) diet, **d** 10,12 CLA diet from day 10 of gestation until parturition (CON/10,12 CLA) or **e** 10,12 CLA diet from 0–10 days of gestation (10,12 CLA/CON). Scale bar is 2 mm. **f** Ductal elongation was measured as the distance from the teat to the furthest-reaching ductal terminus. **g** Ductal area was measured as the polygonal area of the ductal network. **h** Total branch point number was determined and expressed relative to ductal area. *P < 0.05 vs CON within an age group (n = 4–9/age group/diet). Panels **f - h** share the same legend

We next determined the effect of maternal CLA intake during gestation on morphological development of the mammary glands in peripubertal female offspring. Branchpoint density in the mammary glands of females born to dams fed 10,12 CLA for the first half of gestation was significantly increased at both 35 and 55 day of postnatal age (*P* < 0.05, Fig. 2a−e, h). Ductal elongation was unaffected by maternal CLA intake (Fig. 2f). At 35 days of age, area of the mammary ductal network in females born to dams fed 10,12 CLA for the first half of gestation was reduced (*P* < 0.05, Fig. 2g), whereas at 21 or 55 days of age it was unaffected by any of the maternal dietary treatments (*P* > 0.05, Fig. 2g). There was no effect of maternal CLA intake on the rate of epithelial proliferation in the mammary glands at 55 days of age (*P* > 0.05; Supplementary Fig. 1), which may reflect the timing of the short labeling period relative to the morphological changes observed.

**Fig. 3:**
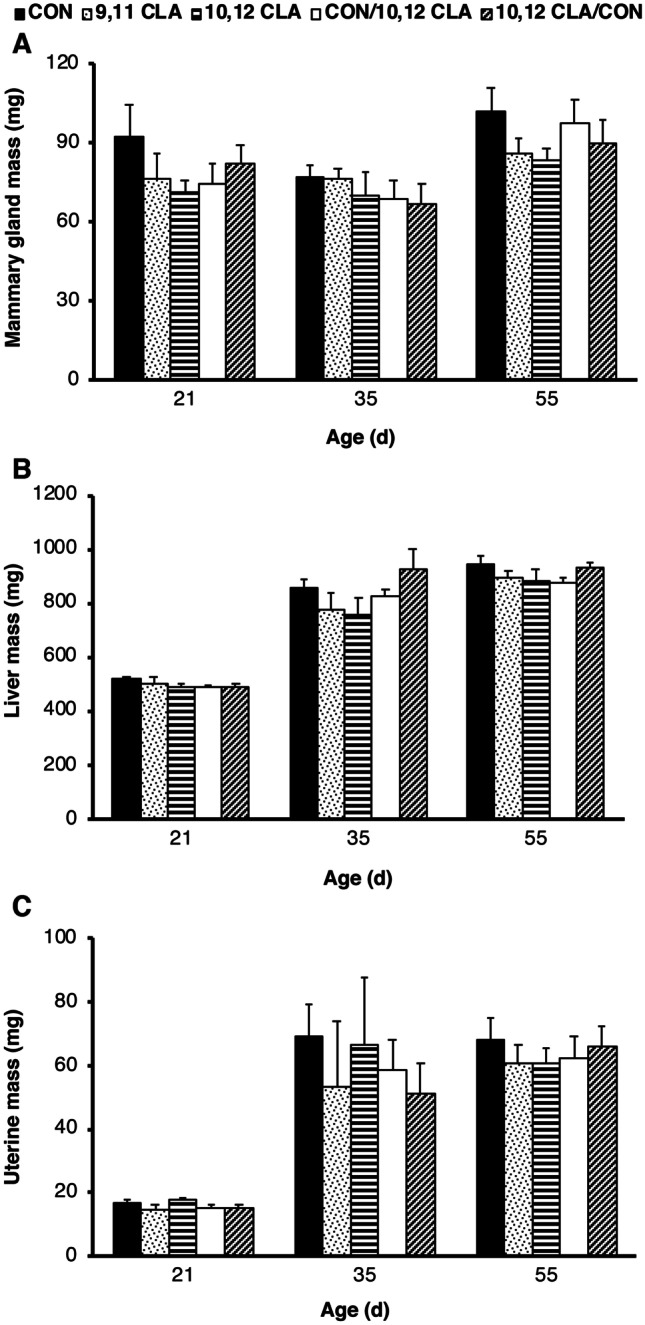
Exposure to 10,12 CLA during early in utero development did not affect postnatal organ growth in Experiment 1. Wet organ mass of **a** lymph-node free inguinal mammary gland, **b** liver, and **c** uterus from 55-day old female mice born to dams fed the control (CON) diet, the *cis*-9, *trans*-11 conjugated linoleic acid (9,11 CLA) diet, the *trans*-10, *cis*-12 conjugated linoleic acid (10,12 CLA) diet, the control diet from day 0–10 then 10,12 CLA diet from day 10 of gestation until parturition (CON/10,12 CLA), or the 10,12 CLA diet from 0–10 days of gestation and control diet from day 10 of gestation until parturition (10,12 CLA/CON). Data are means ± SEM (n = 4–9/age group/diet). Panels **a** − **c** share the same legend

We measured the mass of the mammary glands and liver in female offspring at necropsy given that postnatal consumption of 10,12 CLA reduces adiposity [14] and promotes hepatic lipid accumulation [31]. There was no effect of maternal diet on mammary gland (*P* > 0.05, Fig. 3a), liver (*P* > 0.05, Fig. 3b) or uterine mass (*P* > 0.05, Fig. 3c).

### Experiment 2—Hormone Responsiveness of the Mammary Glands in OVX Female Offspring Born to Dams Fed CLA During Gestation

**Fig. 4:**
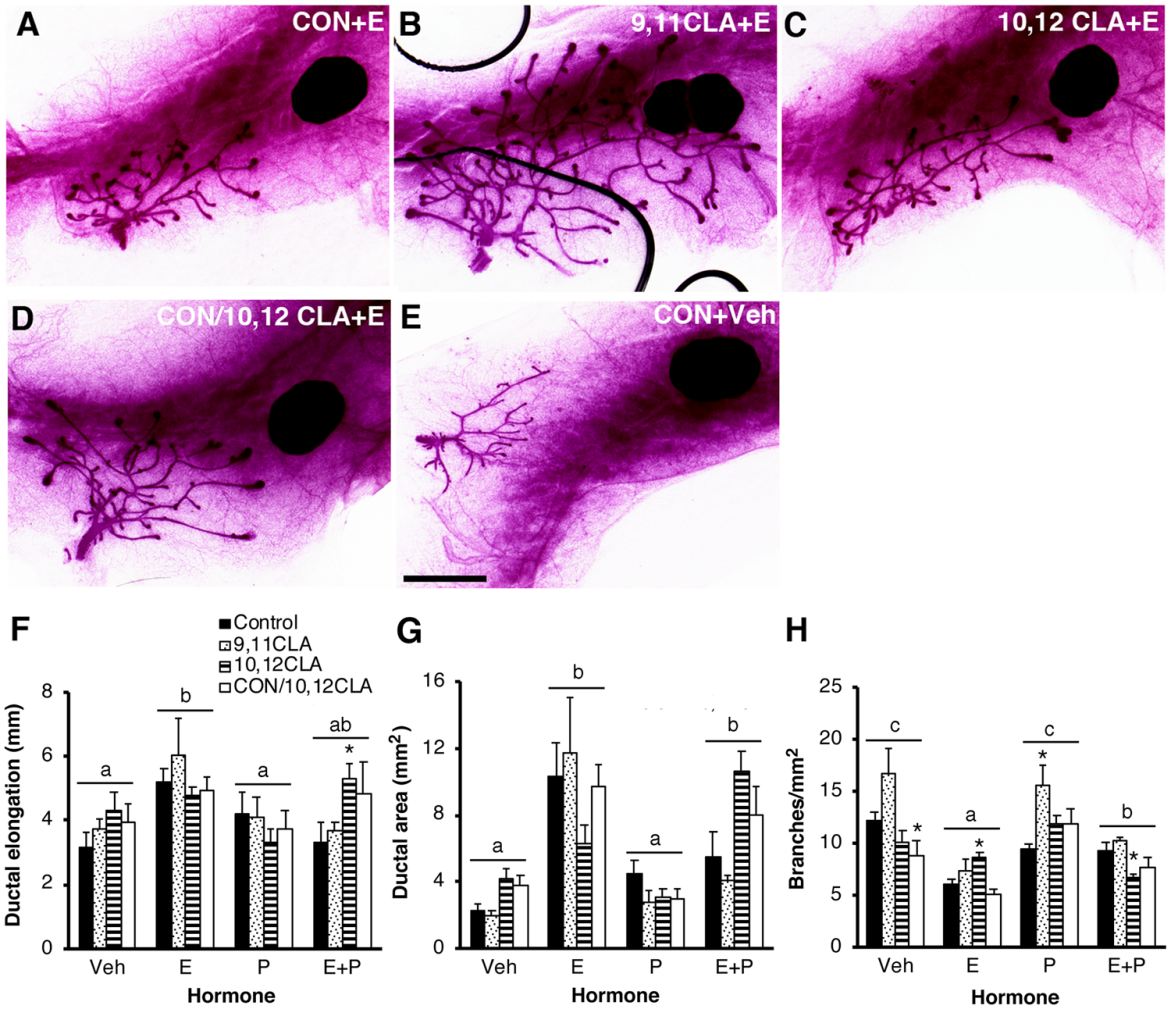
Exposure to 10,12 CLA during in utero development modified mammary gland hormone responsiveness in postnatal, ovariectomized mice in Experiment 2. **a** − **e** Whole mounts of mammary glands from 33-day old female mice exposed via the maternal diet to either: **a** and **e** the control (CON) diet, **b** the *cis*-9, *trans*-11 conjugated linoleic acid (9,11 CLA) diet, **c** the *trans*-10, *cis*-12 conjugated linoleic acid (10,12 CLA) diet or **d** the 10,12 CLA diet only from day 10 of gestation until parturition (CON/10,12 CLA). Mice were ovariectomized at weaning (21 days of age) and implanted with a slow-release pellet at 28d of age containing either **e** the cholesterol vehicle (Veh), 17β-estradiol (E), progesterone (P) or E + P. Scale bar is 2 mm. **f** Ductal elongation was measured from the teat to the furthest-reaching ductal terminus. **g** Ductal area was the polygonal area of the ductal network. **h** Total branch point number was determined and expressed relative to ductal area. *P < 0.05 vs CON within a hormone treatment group. Hormone treatments without a common letter are different, *P* < 0.01 (n = 4–8/hormone/diet). Panels **f** − **h** share the same legend

We next examined the effects of CLA exposure in utero on hormone responsiveness of the mammary glands postnatally. As expected, there was a significant main effect of hormone treatment on ductal elongation (*P* < 0.01; Fig. 4a−f), ductal area (*P* < 0.0001; Fig. 4g) and branchpoint density (*P* < 0.0001; Fig. 4h). Among the OVX mice that were only treated with Veh, those offspring born to dams fed 10,12 CLA during the second half of gestation had reduced branchpoint density compared to females born to control-fed dams (*P* < 0.05, Fig. 4h). Conversely, branchpoint density among E-treated OVX mice was increased in females born to dams fed 10,12 CLA throughout gestation (*P* < 0.05, Fig. 4h). Females that were exposed to 9,11 CLA in utero*,* followed by OVX and treatment with P, had greater branchpoint density compared to P-treated mice born to control-fed dams (*P* < 0.05, Fig. 4h). Amongst OVX mice treated with E + P, females exposed to 10,12 CLA throughout gestation had increased ductal elongation (*P*  < 0.05, Fig. 4f), increased ductal area (*P*  < 0.05, Fig. 4g) and decreased branchpoint density (*P*  < 0.05 vs. control, Fig. 4h). There was no effect of maternal CLA intake during gestation on mammary epithelial cell proliferation at 33 days of age in OVX mice treated with E + P (*P*  > 0.05, Supplementary Fig. 2), likely reflecting the timing of EdU labeling relative to the morphological response.

**Fig. 5:**
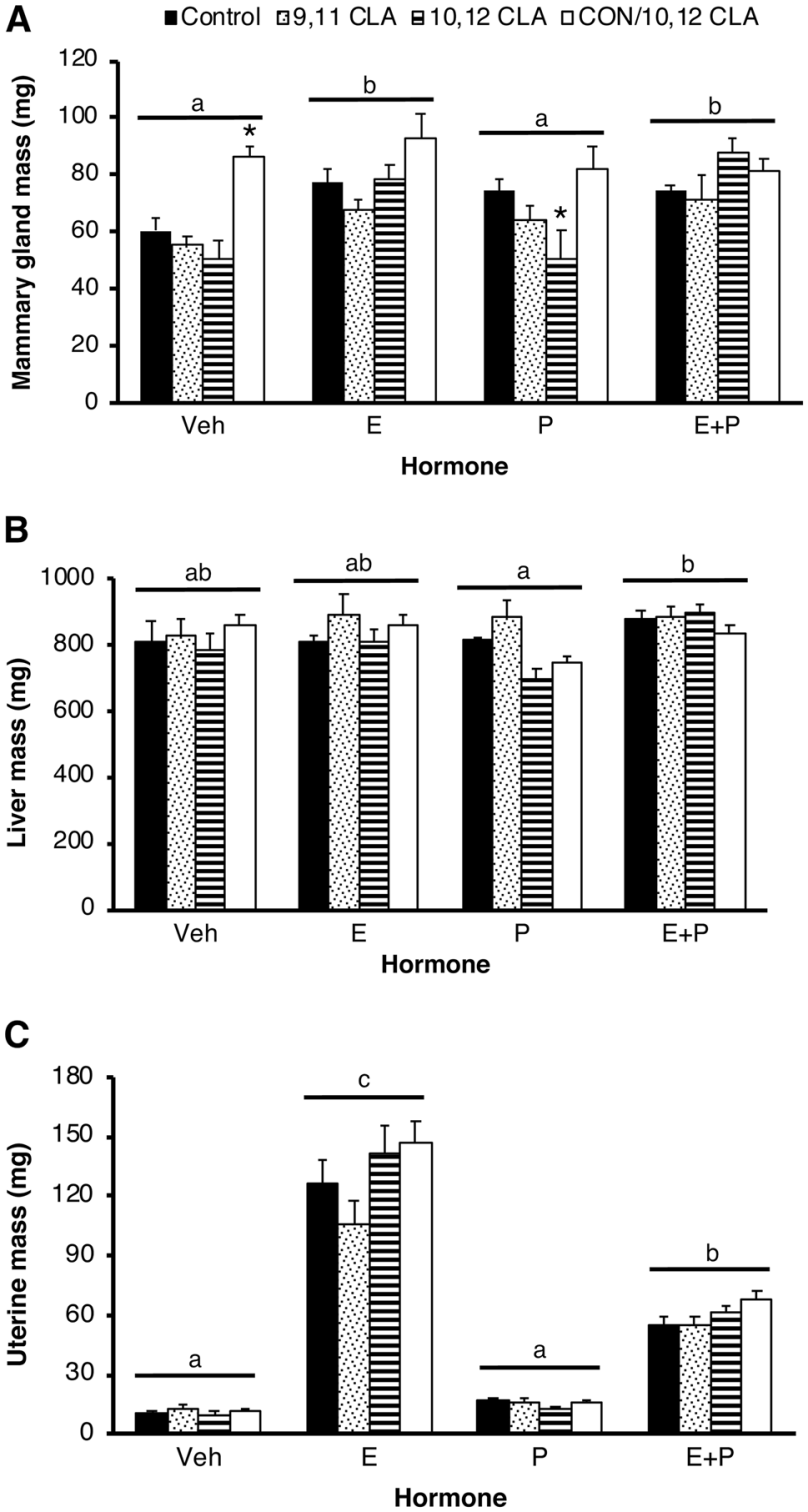
In utero exposure to 10,12 CLA from day 10 of gestation until parturition, or 10,12 CLA throughout development, modified mammary gland mass in postnatal ovariectomized (OVX) non-hormone treated females in Experiment 2. Wet organ mass of **a** lymph-node free inguinal mammary gland, **b** liver, and **c** uterus from 33-day old female mice exposed via the maternal diet to either the control (CON) diet, the *cis*-9, *trans*-11 conjugated linoleic acid (9,11 CLA) diet, the *trans*-10, *cis*-12 conjugated linoleic acid (10,12 CLA) diet or the 10,12 CLA diet only from day 10 of gestation until parturition (CON/10,12 CLA). Mice were OVX at weaning (21 days of age) and implanted at 28 days of age with a slow-release pellet containing the cholesterol vehicle (Veh), 17β-estradiol (E), progesterone (P), or E + P. Data are means ± SEM. *P < 0.05 vs CON within a hormone treatment group. Hormone treatments without a common letter are different, *P* < 0.01 (n = 4–8/hormone/diet). Panels **a**-**c** share the same legend

There were significant main effects of both maternal diet and hormone treatment, and an interaction between maternal diet and hormone treatment, on mammary gland mass (*P*  < 0.05). Amongst OVX mice not treated with hormones, offspring from dams fed 10,12 CLA for the second half of gestation had heavier mammary glands compared to those born to control-fed dams, while offspring from dams fed 10,12 CLA throughout gestation had smaller mammary glands (*P*  < 0.05 vs. control, Fig. 5a). Liver mass was unaffected by in utero exposure to different CLA diets (*P*  > 0.05, Fig. 5b), though there was a main effect of hormone treatment (*P*  < 0.01; Fig. 5b). Uterine mass was also unaffected by in utero exposure to different CLA diets (*P*  > 0.05, Fig. 5c) but, as expected, was increased by E treatment (*P*  < 0.0001), with a smaller increase induced by E + P (*P*  < 0.0001, Fig. 5c).

### Experiment 3—Hormone Responsiveness of the Mammary Glands in Sexually-Mature Female Mice Fed CLA

**Fig. 6:**
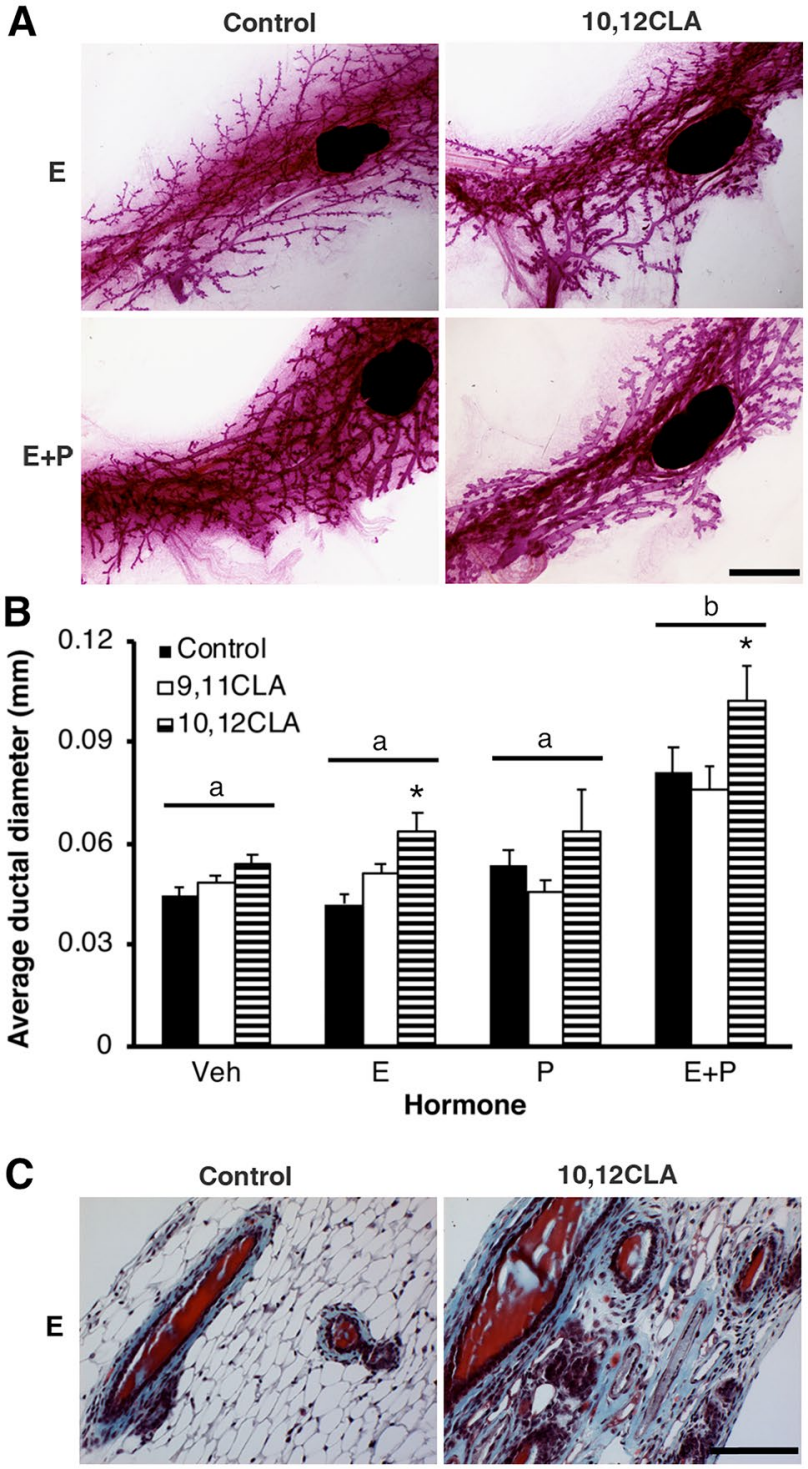
Dietary 10,12 CLA increased ductal diameter in sexually mature female mice administered estrogen in Experiment 3. Sexually mature female mice were fed either the control diet, the *cis*-9, *trans*-11 conjugated linoleic acid (9,11 CLA) diet, or the *trans*-10, *cis*-12 conjugated linoleic acid (10,12 CLA) diet for 28d before ovariectomy (OVX). Commencing 7 days after OVX, mice were injected daily with corn oil vehicle (Veh), 17β-estradiol (E), progesterone (P), or E + P for 4 days. **a** Whole mounts of mammary glands from female mice fed either the control diet or the 10,12 CLA diet and injected with either E, or E + P. Scale bar is 2 mm. **b** Average thickness of ducts in the #4 inguinal mammary glands. Data are means ± SEM. *P < 0.05 vs Control within a hormone treatment group. Hormone treatments without a common letter are different, *P* < 0.0001 (n = 4–13/hormone/diet). **c** Sections of mammary glands from mice fed either the control or 10,12 CLA diet and treated with E, stained with Gomori’s trichrome. Scale bar is 50 μm

**Fig. 7:**
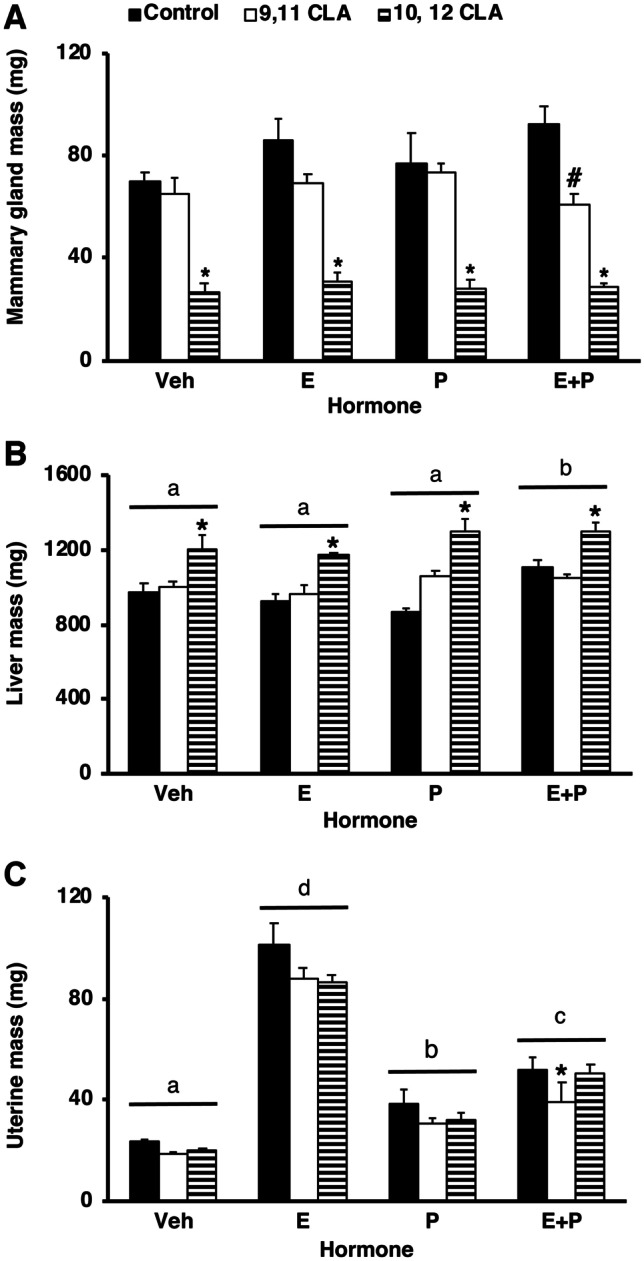
Dietary 10,12 CLA decreased mass of the mammary glands, and increased liver mass, in ovariectomized (OVX) sexually-mature female mice administered estrogen and progesterone (P) in Experiment 3. Wet organ mass of **a** lymph-node free inguinal mammary glands, **b** liver, and **c** uterus from sexually mature female mice fed the control diet, the *cis*-9, *trans*-11 conjugated linoleic acid (9,11 CLA) diet, or the *trans*-10, *cis*-12 conjugated linoleic acid (10,12 CLA) diet for 28d before OVX. Commencing 7d after OVX, mice were injected daily with corn oil vehicle (Veh), 17β-estradiol (E), progesterone (P), or E + P for 4d. Data are means ± SEM. *P < 0.05 vs Control within a hormone treatment group (n = 4–13/hormone/diet). Panels **a**-**c** share the same legend

Given our findings from Experiments 1 and 2, we also determined the effects of exogenous ovarian steroids on the mammary glands of OVX sexually-mature female mice fed a CLA-supplemented diet. Average ductal diameter was increased in mice fed 10,12 CLA then treated with either E or E + P, (*P*  < 0.05, Fig. 6a, b). Analysis of trichrome-stained histological sections revealed that the increased ductal diameter reflected an increase in the cellularity of the periductal extracellular matrix (ECM, Fig. 6c). Given the extracellular environment can also reflect altered invasion by immune cells, we also localized the distribution of F4/80 macrophages, for which there was no difference in their density between E-treated control- or 10,12 CLA-fed mice (*P*  > 0.2, Supplementary Fig. 3). Mammary gland mass was unaffected by hormone treatment (*P*  > 0.1) but was reduced in response to dietary 10,12 CLA (*P*  < 0.05, Fig. 7a). Liver mass was increased in response to dietary 10,12 CLA (*P*  < 0.0001, Fig. 7b), irrespective of hormone treatment, and was also increased by treatment with E + P (*P*  < 0.05, Fig. 7b). As expected, uterine mass was increased by P, E + P and E treatment (*P*  < 0.0001, Fig. 7c). Mass of both the mammary gland and uterus was reduced in mice fed 9,11 CLA and administered E + P (*P*  < 0.05, Fig. 7a, c).

## Discussion

### CLA Modifies the Mammary Response to E

Our results across several independent experiments highlight an ability for dietary CLA, either in utero or during postnatal development, to modulate responsiveness of the mammary epithelium to E, either alone or in combination with P. Specifically, maternal intake of 10,12 CLA during the first half of gestation increased the density of mammary duct branchpoints in 35 and 55 day old female offspring during the allometric phase of ductal development that normally occurs in response to ovary-derived E [32, 33]. In a similar way, females exposed to 10,12 CLA in utero then OVX and administered E in later life also displayed increased branchpoint density. Finally, diameter of the mammary ducts was increased in OVX sexually-mature females fed 10,12 CLA and then administered either E or E + P.

There are several arguments to implicate the microenvironment of the mammary stroma in these responses, which aligns with our finding herein that adult OVX females fed 10,12 CLA and treated with E had increased cellularity of the periductal stroma. Indeed, dietary 10,12 CLA modifies the adipose microenvironment of the mammary glands in postnatal mice, including by stimulating lipolysis and atrophy in mammary adipocytes [34], thereby increasing infiltration of immune cells in association with inflammation and adipokine synthesis [35], and by activating IGF-dependent growth [15]. In a similar way, the stromal microenvironment plays an important role in mediating responses by the adjacent mammary epithelium to E [36], including through the synthesis of various extracellular matrix molecules [37], the synthesis and activity of a range of growth factors [15, 38, 39], or by changing the local fatty acid profile [40]. Further studies are needed to establish whether these axes converge on the stroma during co-signaling by E and CLA to the mammary epithelium—a proposal that is further substantiated by our RNA-sequencing results for the transcriptomic changes induced by either E or CLA [41]. In that context, E alone, consistent with findings by others, stimulated the local synthesis of various EGFR ligands, including amphiregulin [42]. Meanwhile, dietary CLA stimulated the synthesis of stromal EGFR, in addition to increasing the transcription of various EGF signaling components including the EGFR docking protein, Gab2, and the ADAM12 and -17 sheddases that release and activate bound ligands, including amphiregulin [41]. In a separate study, culturing 3T3-L1 adipocytes with 10,12 CLA increased their production of another EGFR ligand, epiregulin [43]. These different lines of evidence here and in the published literature support the potential for a local growth regulatory loop whereby E could increase the local biosynthesis of stroma-derived EGF-related ligands, including amphiregulin, that could then stimulate surrounding stromal cells, through their CLA-induced EGFR, to increase epithelial growth and morphogenesis.

### Differential Responses to CLA In Utero

We also recorded that the mammary gland responses following exposure to CLA in utero were distinct from those following postnatal exposure. At the systemic level, feeding 10,12 CLA to postnatal mice induces metabolic dysregulation manifesting as lipoatrophy [34], hepatic steatosis [31], adipose inflammation [44] and hyperinsulinemia [45], whereas 9,11 CLA does not evoke such metabolic responses [46]. These outcomes also vary according to age [47], species [48], and genotype [15]. In this study, consumption of 10,12 CLA by OVX, sexually-mature female mice also reduced mammary gland mass whereas liver mass was increased, consistent with our previous findings [15] and those of others [17, 31, 44]. By contrast, in utero exposure to 10,12 CLA did not alter any of these systemic parameters postnatally. At the same time, development of the mammary glands in response to 10,12 CLA differed according to the timing of exposure. Whereas feeding 10,12 CLA to postnatal OVX females stimulated mammary gland growth independent of E and ESR [15], no such effect was recorded in females born to dams fed 10,12 CLA then subsequently OVX at weaning. This stage-specific differential response to 10,12 CLA stands to reason given the intermediary role of the placenta in modulating lipid transfer to the fetus [49] and the much-lower levels of 10,12 CLA that accumulated in neonates born to dams fed 10,12 CLA relative to levels following postnatal exposure. Of interest, similarities exist between the response to E after in utero exposure to 10,12 CLA and for other in utero exposure models. For example, when a diet high in unsaturated fats was fed to pregnant rats, the mammary glands of their female offspring had more E-sensitive terminal end buds [8, 12] that was linked to elevated maternal circulating estrogens [8]. Similarly, female mice exposed in utero to the environmental estrogen, bisphenol A, subsequently had increased mammary branching [50]. While further mechanistic studies are needed to resolve when and how 10,12 CLA exposure in utero affects postnatal development and E-responsiveness, it is tempting to speculate about a potential common role for the fetal mesenchyme in all models, given that stromal expression of peroxisome proliferator-activated receptor-γ [51] and/or altered ESR expression and function have been implicated across these different systems [12, 15, 51]. Given that elements of the mammary gland microenvironment were also altered epigenetically in response to either bisphenol A [52] or a diet high in polyunsaturated fatty acids [53], any effect of in utero exposure to dietary fats such as 10,12 CLA may also impart transgenerational consequences.

## Conclusions

Our findings support accumulating evidence for a link between the maternal diet and postnatal development of her offspring, including for the mammary glands. We conclude that dietary CLA modifies the mammary gland response to E when exposure occurs during distinct developmental windows, either during in utero development, or postnatally. Further studies are warranted to evaluate the precise timing of these effects and their implications for functional endpoints ranging from lactation success to cancer risk.

## Supplementary Information

Below is the link to the electronic supplementary material.

Supplementary file1 (TIFF 322 KB)

Supplementary file2 (TIFF 1751 KB)

Supplementary file3 (TIFF 12591 KB)

## Data Availability

The datasets generated during and/or analysed during the current study are available from the corresponding author on reasonable request.

## Abbreviations

9,11 CLA: *cis*-9, *trans*-11 Conjugated linoleic acid
10,12 CLA: *trans*-10, *cis*-12 Conjugated linoleic acid
CLA: Conjugated linoleic acid
E: Estrogen
P: Progesterone
OVX: Ovariectomized
Veh: Vehicle
HFD: High-fat diet
TEB: Terminal end bud
ESR: Estrogen receptor
DHA: Docosahexaenoic acid
