# Supplementary figures and images for: In Utero Exposure to trans-10, cis-12 Conjugated Linoleic Acid Modifies Postnatal Development of the Mammary Gland and its Hormone Responsiveness
Source: J Mammary Gland Biol Neoplasia. 2021 Oct 6;26(3):263–76. doi: 10.1007/s10911-021-09499-y (PMC8566432; doi:10.1007/s10911-021-09499-y)

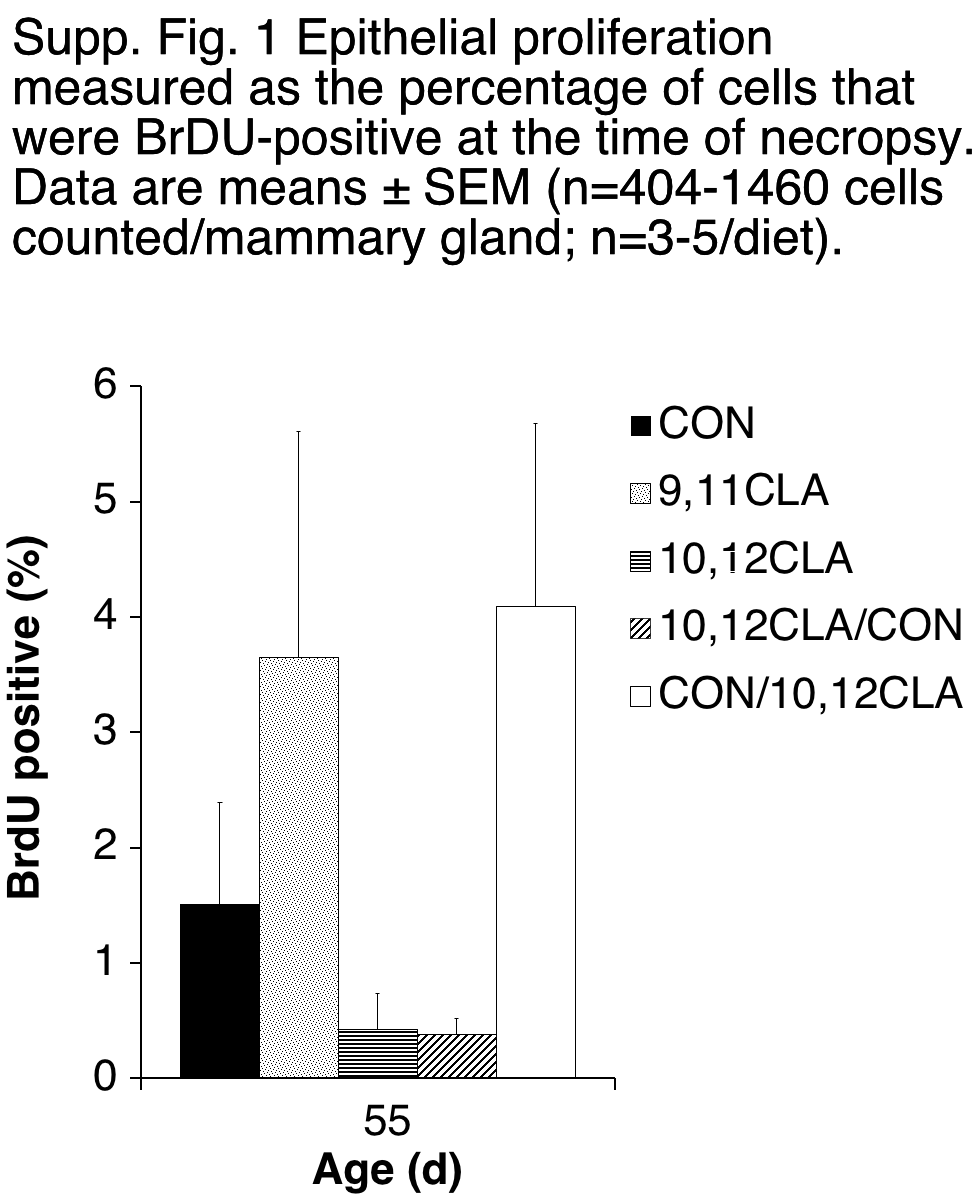

Supplement: Supplementary file 1 — Supplementary file1 (TIFF 322 KB) [file 10911_2021_9499_MOESM1_ESM.tiff]

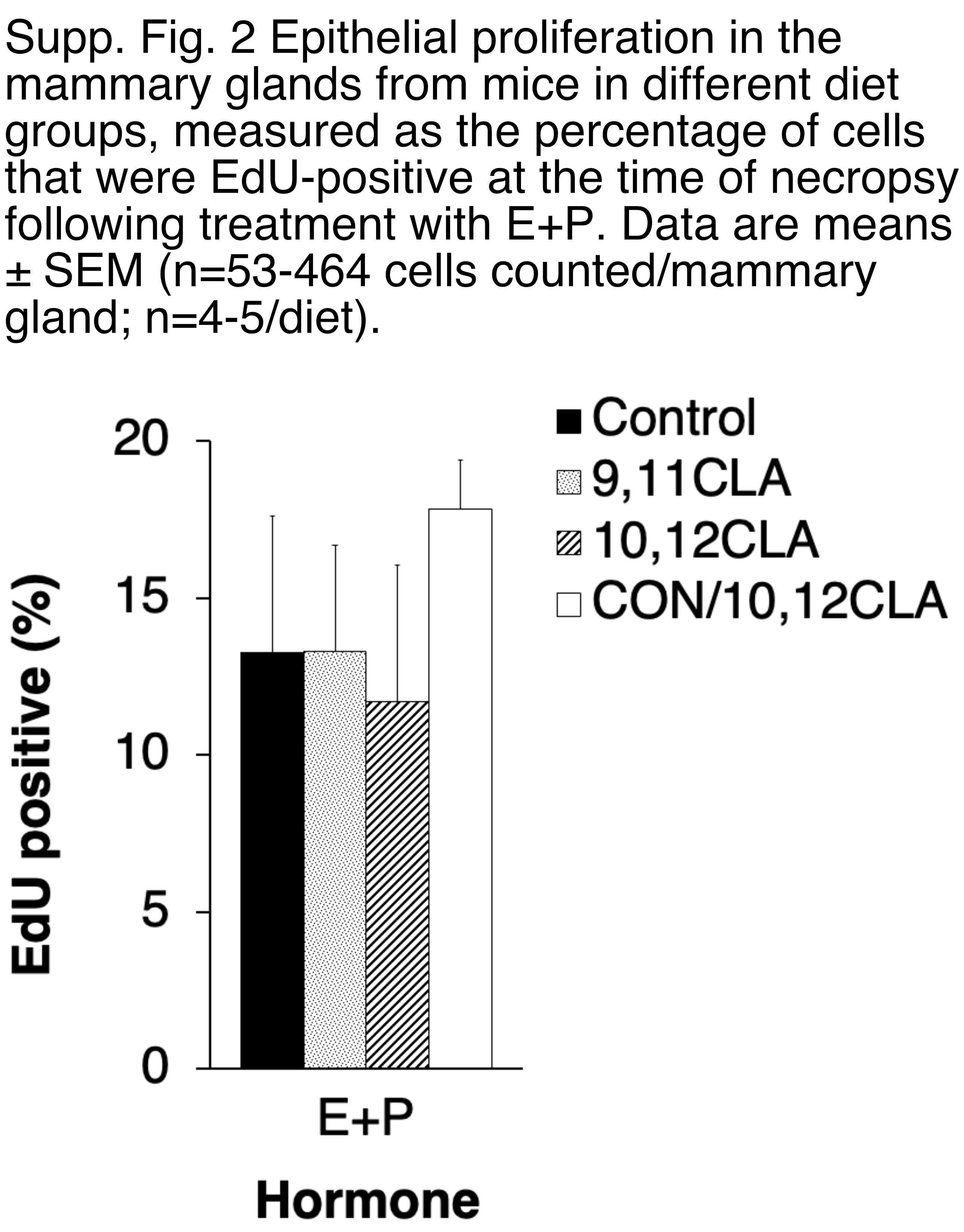

Supplement: Supplementary file 2 — Supplementary file2 (TIFF 1751 KB) [file 10911_2021_9499_MOESM2_ESM.tiff]

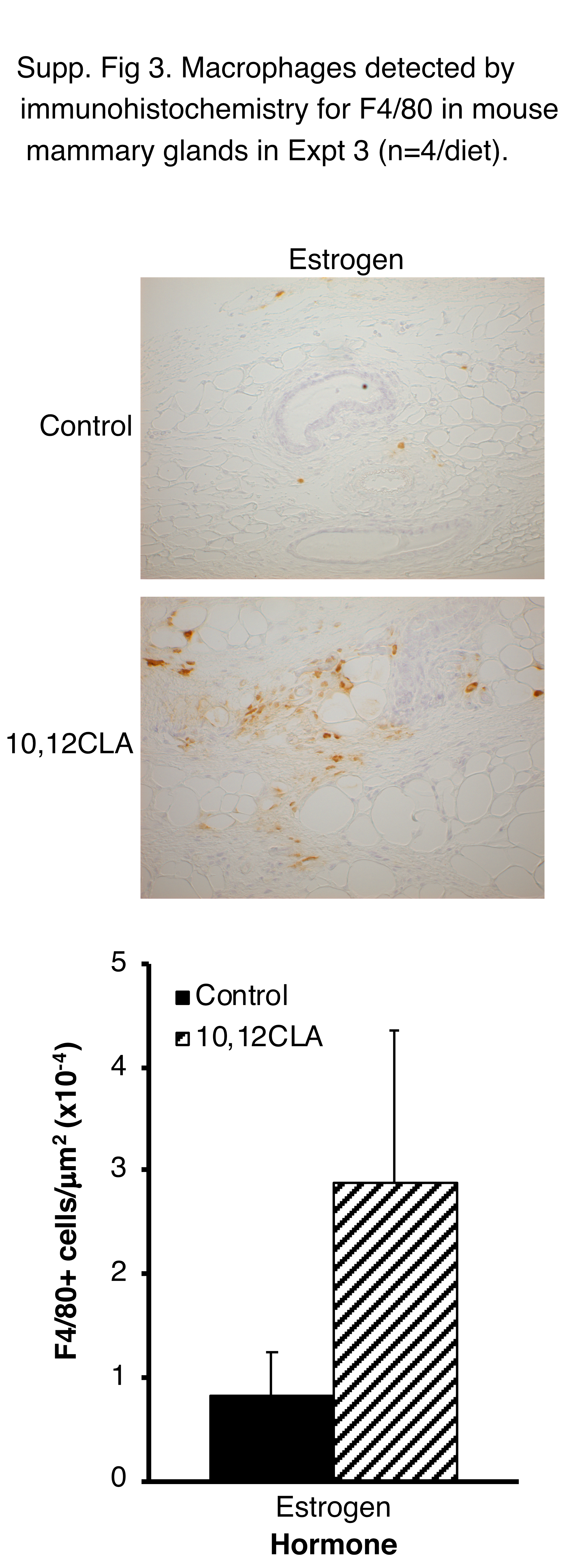

Supplement: Supplementary file 3 — Supplementary file3 (TIFF 12591 KB) [file 10911_2021_9499_MOESM3_ESM.tiff]
